# Retraction Note: Reanalysis of the gene expression profile in chronic pancreatitis via bioinformatics methods

**DOI:** 10.1186/s40001-015-0121-9

**Published:** 2015-03-26

**Authors:** Hang Yuan, Bin Wu, Senlin Ma, Xiaoyu Yang, Lei Yin, Aijun Li

**Affiliations:** Department of the 2nd Special Treatment, Eastern Hepatobiliary Surgery Hospital, Second Military Medical University, No 225, Changhai Road, Shanghai, 200438 China

## Retraction

The Publisher and Editor regretfully retract this article [1] because the peer-review process was inappropriately influenced and compromised. As a result, the scientific integrity of the article cannot be guaranteed. A systematic and detailed investigation suggests that a third party was involved in supplying fabricated details of potential peer reviewers for a large number of manuscripts submitted to different journals. In accordance with recommendations from COPE we have retracted all affected published articles, including this one. It was not possible to determine beyond doubt that the authors of this particular article were aware of any third party attempts to manipulate peer review of their manuscript.

## References

[1] Yuan H, Wu B, Ma S, Yang X, Yin L, Li A (2014). Reanalysis of the gene expression profile in chronic pancreatitis via bioinformatics methods. Eur J Med Res.

